# Expert consensus on clinical trial design guidelines for FLASH radiotherapy

**DOI:** 10.1002/pro6.70098

**Published:** 2026-09-23

**Authors:** Hui Luo, Chen Cheng, Chengliang Yang, Bing Li, Jiandong Zhang, Jinbo Yue, Zhaoyang Lou, Hui Liu, Jing Cai, Hong Ge

**Affiliations:** ^1^ Department of Radiation Oncology The Affiliated Cancer Hospital of Zhengzhou University Zhengzhou China; ^2^ HNHC Key Laboratory of Henan Cancer Hospital Zhengzhou China; ^3^ Department of Strategy and Business The Affiliated Cancer Hospital of Zhengzhou University Zhengzhou China; ^4^ Department of Radiation Oncology Shandong Provincial Qianfoshan Hospital Jinan China; ^5^ Department of Radiation Oncology Shandong Cancer Hospital Jinan China; ^6^ Department of pathology and Pathophysiology School of Basic Medical Sciences College of Medicine Zhengzhou University Zhengzhou China; ^7^ Department of Health Technology and Informatics The Hong Kong Polytechnic University Hong Kong SAR China

**Keywords:** FLASH radiotherapy, clinical trial design, translational application, radiation‐induced normal tissue toxicity, expert consensus

## Abstract

In recent years, ultra‐high dose‐rate FLASH radiotherapy (FLASH‐RT) has emerged as a prominent research focus in radiation oncology. A series of preclinical data indicate that FLASH‐RT can achieve tumor‐killing efficacy that is not inferior to that of conventional radiotherapy (CONV‐RT) while significantly reducing radiation damage to normal tissues. This distinctive tissue‐sparing effect is known as the “FLASH effect.” Rapid progress has been made in the clinical translation and application of FLASH‐RT. Against this backdrop, the China Anti‐Cancer Association Radiation Oncology Committee and the Chinese Medical Doctor Association Radiation Oncology Physician Committee gathered a group of experts and compiled the “Expert Consensus on Clinical Trial Design Guidelines for FLASH Radiotherapy” based on the latest domestic and international research advancements. This document summarizes the latest advances in FLASH‐RT and offers practical guidance for its design and translational application in future clinical trials.

## Graphical abstract

• FLASH radiotherapy has emerged as a transformative technology due to the underlying FLASH effect.

• As FLASH radiotherapy moves toward clinical application, establishing standardized trial design principles is critical.

• This expert consensus provides a roadmap for rigorous clinical evaluation of FLASH radiotherapy, accelerating safe translation of this promising technology.

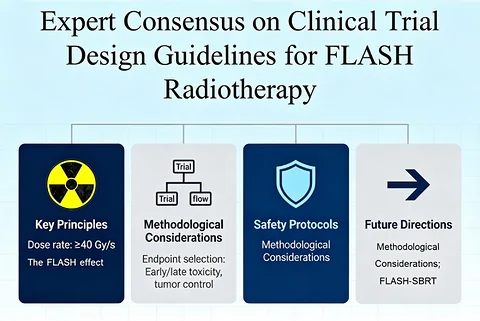

## 1 PURPOSE AND BACKGROUND

Radiotherapy is a cornerstone of cancer treatment and contributes to approximately 40% of cancer cures worldwide, according to the World Health Organization.^1^ However, its effectiveness is intrinsically limited by collateral damage to surrounding normal tissues.^2^ In clinical practice, the need to respect normal tissue tolerance often necessitates compromising the radiation dose delivered to the tumor, thereby limiting therapeutic efficacy and creating a plateau in outcomes for conventional radiotherapy (CONV‐RT).^3^ Ultra‐high dose rate FLASH radiotherapy (FLASH‐RT), generally defined by an average dose rate exceeding 40 Gy/s, has recently emerged as a transformative research direction.^4^ Extensive preclinical studies demonstrate that compared to CONV‐RT, FLASH‐RT can achieve non‐inferior tumor control rates while significantly sparing normal tissues from radiation damage—a phenomenon termed the “FLASH effect.”^4^, ^5^, ^6^, ^7^ This effect has been robustly observed across various radiation modalities, animal species, and tissue sites, generating considerable enthusiasm within the radiation oncology community and driving a surge in translational research.^5^, ^8^, ^9^, ^10^, ^11^ Given this promising preclinical foundation, there is a pressing need to establish robust frameworks for clinical translation. This expert consensus specifically addressed FLASH‐RT and aimed to provide methodological guidance for the design, conduct, and evaluation of clinical trials. By promoting harmonization and standardization, this consensus seeks to facilitate the generation of high‐quality evidence regarding the efficacy and safety of FLASH‐RT, ultimately supporting its rigorous clinical integration and expanding the technological arsenal available for patient care.

## 2 DRAFTING PROCESS

This expert consensus was jointly drafted by experts from the China Anti‐Cancer Association Radiation Oncology Committee and the Chinese Medical Doctor Association Radiation Oncology Physician Committee. The work commenced in September 2025, and a preliminary draft was completed in December 2025. Following revisions involving several clinical experts in China, a consultation draft was finalized in February 2026.

## 3 DEFINITIONS AND TERMINOLOGY

### 3.1 Non‐inferiority trial design

A trial designed to test whether a new treatment method is not inferior to another conventional treatment method. This design is commonly used in clinical studies with objective efficacy endpoints.

### 3.2 Superiority trial design

A trial designed to test whether a new treatment method is superior to another conventional treatment method. Trials using conventional treatments as controls often employ superior designs. The null hypothesis of the superiority trial was that the overall efficacy of the new treatment method (T) was equal to or worse than that of the conventional treatment method (C), whereas the alternative hypothesis was that the overall efficacy of the new treatment method was superior to that of the conventional treatment method. Rejecting the null hypothesis leads to the conclusion that the new treatment method is superior to the conventional treatment method.

### 3.3 Factorial design

A type of experimental design that involves multiple factors (two or more) to investigate the main effects of each factor and the interaction effects between the factors. It is based on completely randomized, randomized block, and Latin square designs.

## 4 KEY CONSIDERATIONS IN TRIAL DESIGN

### 4.1 Participant selection

Participants should meet the requirements of FLASH‐RT. Based on the dosimetric characteristics of FLASH‐RT, potentially eligible populations should be screened to further define the target population. The inclusion and exclusion criteria should be relevant to the hypothesized mechanism of action of the FLASH‐RT. Appropriate participants should be selected by considering the mechanism of action of FLASH‐RT as well as data from non‐clinical studies and early‐phase clinical trials.

Generally, strict inclusion and exclusion criteria are designed to enhance the internal validity of the study itself, whereas broad enrollment and exclusion criteria can improve the generalizability or external validity of the study findings. During the design phase, patient selection should involve collaboration among clinical researchers, radiation physics experts, and radiation technology experts to assess the feasibility of resources and operations and balance the study's internal validity and external generalizability. Based on the study objectives, demographic and disease characteristics should be considered during selection, with a focus on the following factors:

#### 4.1.1 Definition of FLASH‐RT

The definition of FLASH‐RT is primarily based on the article “Mechanisms, challenges and opportunities for FLASH‐RT in cancer” published in *Nature Reviews Cancer* by Professor Marie‐Catherine Vozenin, an international pioneer in the field of FLASH‐RT.^4^ Its core definition can be summarized as follows: an advanced radiotherapy technique that delivers a high dose of radiation in an extremely short period of time (typically < 200 milliseconds) at an ultra‐high dose rate (average dose rate ≥40 Gy/s, instantaneous dose rate ≥100 Gy/s) far exceeding that of CONV‐RT. Therefore, the goal is to effectively kill tumors while significantly reducing the damage to normal tissues. Its core characteristics are as follows.

- Ultra‐High Dose Rate. The average dose rate is ≥40 Gy/s, typically hundreds to thousand times higher than that of CONV‐RT (approximately 0.01–0.1 Gy/s).
- Extremely Short Irradiation Time. The entire treatment process is typically completed within a few hundred milliseconds and delivered as one or several extremely short pulses.
- High Single‐Fraction Dose. The dose delivered per treatment session is much higher than the dose per fraction in CONV‐RT (typically ≥5 Gy, or even up to tens of Gy).
- Key Biological Phenomenon: FLASH Effect. This is the theoretical foundation of FLASH‐RT, referring to a unique differential effect observed under ultra‐high dose rate irradiation: Its tumor‐killing efficacy is similar or non‐inferior to that of CONV‐RT, and it is particularly effective against hypoxic tumors. For normal tissues, it produces unexpected protective effects, and damage is significantly reduced.

Furthermore, current preclinical studies on FLASH‐RT predominantly employ a single high‐dose irradiation. An excessively high single dose may exceed the self‐repair capacity of the tissue, leading to exacerbated normal tissue damage, thereby masking the radioprotective effect of FLASH in reducing normal tissue injury. Drawing on the technical experience of CONV‐RT is a key strategy for achieving the clinical translation of FLASH‐RT and maximizing its therapeutic potential. Therefore, to design FLASH‐RT protocols, hypofractionated FLASH‐RT or FLASH‐Stereotactic Body Radiation Therapy (FLASH‐SBRT) is recommended.^12^ This approach, based on delivering ablative doses to tumors at ultrahigh dose rates, protects normal tissues through the FLASH effect generated by each hypofraction and utilizes the intervals between fractions to allow normal cells to repair damage, thereby achieving optimal normal tissue preservation.

#### 4.1.2 Ethical considerations

Despite decades of research, the precise biological mechanisms that underpin the effects of FLASH‐RT remain incompletely understood, and many crucial questions about late toxicity, carcinogenesis, immunomodulation, and organ‐specific effects remain unanswered. This uncertainty places FLASH‑RT trials in a distinct ethical category: they are not simply testing a refinement of an established technology but are exploring a fundamentally different radiobiological regimen whose long‑term consequences are largely unknown.

The core ethical challenge is that a valid informed consent requires disclosure of material risks; however, for FLASH‑RT, some risks are unknown and perhaps even unknowable at the present time. Patients must be told not only what is known (e.g., that FLASH‑RT has not been approved by the government regulatory authorities and that its acute side‑effect profile is expected to resemble that of CONV‐RT) but also what is not known. Several specific categories of biological uncertainty warrant an explicit discussion during the consent process.

- Uncertain long‐term safety. CONV‐RT is associated with a well‐documented risk of second malignant neoplasm (SMNs) that has persisted for decades. It is not yet known whether FLASH‐RT reduces, increases, or eliminates the risk of SMNs and other late effects, such as cardiovascular toxicity, fibrosis, or endocrinopathies. Until comparative long‐term data become available, this uncertainty must be communicated to patients enrolling in FLASH‐RT trials.
- Unknown mechanisms of normal tissue sparing. More than a dozen hypotheses have been proposed to explain the FLASH effect, including oxygen depletion, radical recombination, mitochondrial preservation, and immune modulation; however, none have been definitively validated, and some may produce unexpected biological consequences in specific organ systems. For example, the extent to which FLASH‑RT spares or disrupts male reproductive function is unknown, and its interaction with the immune system appears to be a “double‑edged sword” that is not fully characterized.
- Technical and dosimetric variability. FLASH‐RT requires specialized equipment and real‐time dosimetry systems that are still under development. Different beam modalities (e.g., electrons vs. protons) and delivery parameters can produce different biological effects, implying that early trial results may not be generalizable across platforms.
- In addition to disclosing these uncertainties, the consent process must avoid therapeutic misconceptions: the belief that the trial treatment is likely to provide direct clinical benefits. FLASH‐RT remains experimental, and early phase trials are designed primarily to assess feasibility and toxicity rather than therapeutic efficacy. This distinction should be reinforced throughout the discussion on consent.
- Given the gaps in the current knowledge, an adaptive and longitudinal approach to obtaining informed consent is ethically advisable. Patients should be re‑consented whenever new safety data emerge during the trial, especially if those data suggest an unforeseen risk. Furthermore, independent oversight bodies should require that consent forms for FLASH‐RT trials prominently state the degree of biological uncertainty and the investigational nature of the treatment, using language that is understandable by patients with limited scientific background.
- Finally, it is important to recognize that prematurely rushing FLASH‑RT into widespread clinical use before the magnitude of its long‑term risks is understood “would be harmful not only for the involved patients but for the entire field of FLASH.” Ethical conduct of FLASH‑RT trials therefore requires not only robust informed consent processes but also appropriate patient selection, such as limiting enrollment to adult patients with adequate life expectancy to permit meaningful follow‑up for late effects, as recommended by recent international consensus.

#### 4.1.3 Age considerations

FLASH‐RT remains in the early stages of clinical exploration (primarily Phase I and Phase I/II trials). The majority of FLASH‐RT clinical trials initiated globally (including FAST‐01 and FAST‐02 in the United States; FLASH‐HN001 in China; and IMPULSE, Flash‐Skin 1, and LANCE in Switzerland) predominantly enroll adult patients.^13^, ^14^, ^15^, ^16^, ^17^, ^18^ The primary objective is to evaluate safety and tolerability, with preliminary efficacy as a secondary consideration. Therefore, the enrollment criteria (including age) must be strict and specific.

A. Adult Trials. The most common age range is 18–80 years (or 85 years). This is primarily based on safety considerations; the physiology and organ functions of adults are relatively stable, facilitating the evaluation of treatment‐related toxicities. Elderly patients may have more comorbidities, so the upper age limit is typically restricted to individuals who can tolerate treatment (usually requiring a good performance status, such as ECOG 0–2). Scientific considerations: Children undergoing growth and development are excluded because the effects of radiation on children are fundamentally different from those on adults, and ethical reviews of pediatric studies are more stringent.
B. Trials that target specific populations. Elderly Patient Trials: These may specifically focus on certain tumor types (e.g., inoperable lung cancer or skin cancer in elderly patients). The lower age limit can be adjusted on the basis of the patient's performance status and organ function. In principle, the upper age limit should not exceed 80 years old. A disproportionately high enrollment of younger participants could lead to bias in the results; therefore, this should be addressed through stratification and/or considered in the data analysis to avoid age‐related bias. Pediatric Trials: Currently, such trials are extremely rare. Although FLASH‐RT holds great potential for protecting normal tissues in pediatric oncology, children have extremely high radiation sensitivity and concerns regarding their long‐term development. Therefore, dedicated pediatric clinical trials will only be initiated after sufficient adult safety data have been obtained.

#### 4.1.4 Disease severity

Disease severity can affect efficacy evaluation. Therefore, patients should be selected based on the research objectives, and the severity of the condition among the enrolled patients should be as consistent as possible. Significant differences in disease severity may hinder the ability to accurately assess the therapeutic effects of FLASH‐RT.

#### 4.1.5 Classification of FLASH‐RT

The FLASH effect is a biological phenomenon and, theoretically, its occurrence does not depend on the type of radiation. As long as irradiation is delivered at a sufficiently high dose rate (≥40 Gy/s), this normal tissue protection effect can be triggered by photons, electrons, protons, or heavy ions. However, owing to the differences in the physical characteristics of various radiation types, such as penetrability, Bragg peak, and RBE, the technical difficulty of achieving FLASH irradiation, clinical application scenarios, and ultimate comprehensive biological effects can vary significantly. Therefore, classification by radiation type has a clear physical and clinical significance.

- Electron FLASH‐RT. Currently, this is the most extensively studied and rapidly advancing clinical research, primarily utilizing high‐energy electron beams.
- Photon FLASH‐RT. This is the most advanced preclinical study in China that primarily utilizes high‐energy X‐rays.
- Proton FLASH‐RT. Currently, this is one of the most anticipated directions with significant potential.
- Heavy ion FLASH‐RT. This mainly refers to treatments using heavier particles, such as carbon ions, which are currently in a very early exploratory stage.
- Very high‐energy electron (VHEE) FLASH‐RT. VHEE beams have a higher energy, providing sufficient penetration to treat deep‐seated tumors, making them a promising direction for the future.

#### 4.1.6 Exclusion criteria

The exclusion criteria for FLASH‐RT clinical trials should be strict and specific and should be determined by their cutting‐edge nature, the characteristics of the trials, and the primary goal of prioritizing safety. The design of the excluded populations should aim to maximize participant safety and ensure the scientific validity and evaluability of the study results. The exclusion criteria include:

- Poor Performance Status. Eastern Cooperative Oncology Group score of >2 or a Karnofsky score of <70%, indicating that the patient is bedridden for a significant portion of the day or is unable to care for themselves.
- Major Organ Dysfunction. Severe abnormalities such as bone marrow suppression (white blood cells, platelets, and hemoglobin below certain values), liver function (significantly elevated transaminase levels), and kidney function (excessively low creatinine clearance).
- Conflicting Prior Treatment History. The inclusion of prior radical radiotherapy at the same site reduces normal tissue tolerance and poses a high risk of cumulative radiation damage.
- Active Second Primary Cancer (other than the target tumor in the trial).
- Pregnant or Lactating Women.
- Patients with Very Short Life Expectancy. For example, those with a life expectancy of < 3 months or < 6 months.
- Patients with Uncontrolled Severe Comorbidities. Including patients with uncontrolled active infections, myocardial infarction within the last 6 months, unstable angina, congestive heart failure (NYHA class III–IV), and uncontrolled severe arrhythmias.
- Tumor Type/Location Mismatch. Each trial should have specific indications, and tumors outside these indications should be excluded.
- Inability to Safely Separate the Planned Irradiation Field (target volume) from Critical Organs. For example, when a tumor is directly adjacent to or encases organs that are highly sensitive to radiation with severe consequences of damage, such as the spinal cord, optic nerve, or intestines, these organs cannot be adequately spared through technical means (e.g., beam angles).
- Excessive or Diffuse Target Volume. For instance, if the planned tumor volume exceeds a predefined limit.
- Inability to Cooperate with Treatment or Complete Follow‐up. Inability to maintain a stable position during treatment due to psychiatric illness, cognitive impairment, or severe pain.
- Inability to Provide Informed Consent.
- Others. Additionally, participants with a known history of severe or idiosyncratic adverse reactions to radiotherapy should be excluded. The basic criteria for dropout, withdrawal, elimination, and termination can be found in Good Clinical Practice (GCP) guidelines and should be established based on the characteristics of the FLASH‐RT beam used in the trial and the specific phase of the clinical study.

### 4.2 Types of trial design

The type of trial design should be determined based on the primary research objectives. For clinical trials investigating new radiotherapy technologies that need to be compared with CONV‐RT techniques with established clinical efficacy, a non‐inferiority trial design with active controls may be employed. A Phase I trial, or single‐arm efficacy trial design, is recommended for new radiotherapy techniques without clear evidence of clinical effectiveness. Alternatively, a factorial design may be considered to investigate FLASH‐RT both as a standalone treatment and in combination with CONV‐RT.

### 4.3 Setting of control groups

An appropriate control group should be selected based on different research objectives and existing clinical treatment standards. The control groups should primarily consist of comparisons with CONV‐RT or comparisons between different FLASH‐RT dose groups. Caution should be exercised when selecting a positive control group for non‐inferiority trials. The general principle for selection is that a suitable positive control should be widely used, whose efficacy has been confirmed and quantified by evidence from well‐designed superiority trials, and that can maintain its original efficacy in the new positive control trial. In principle, all clinical trials should include a control group, and blinding should be maintained as much as possible. The trial design should also fully consider advances in relevant clinical and statistical practices.

### 4.4 Blinding

Blinding ensures that one or more parties involved in the trial are unaware of the treatment the patient receives, including the blinding of the participants and evaluators, among others. The implementation of blinding is a crucial part of ensuring that a clinical trial is conducted according to plan, as it helps reduce human confounding factors in the study. Without blinding, human expectations could influence the results.

In principle, the greater the extent of blinding, the lower the risk of bias, and the higher the quality of the study. Trials using triple‑blind or double‑blind designs are superior to those with single‑blind or no blinding. However, if practical difficulties make it impossible to blind a specific party, the strictest possible blinding design should be chosen based on actual circumstances (Table 1).

**TABLE 1:** Blinding and Definitions.

Blinding Method	Definition
Open‐label	The participants, clinical evaluators, and data statisticians are all aware of the treatment group to which the patient is assigned.
Single‐blind	Only the participants are unaware of the treatment group to which they are assigned.
Double‐blind	Neither the participants nor the evaluators are aware of the treatment group to which the patient is assigned.
Triple‐blind	The participants, clinical evaluators, and data statisticians are all unaware of the treatment group to which the patient is assigned.

### 4.5 Randomization

Randomization and blinding are important methods for reducing trial bias. All clinical trials should employ appropriate randomization methods, including simple, block, and stratified approaches.

### 4.6 Data collection

Collecting data on dosimetric characteristics, safety, adverse events, and compliance with FLASH‐RT is essential. To ensure data quality and integrity, a feasible, scientific, and comprehensive Case Report Form (CRF) should be designed. The CRF must possess the following characteristics: it should be detailed and informative to meet research needs; it should be validated and user‐friendly; its content should be standardized with clearly defined indicators; and a pilot test is recommended to ensure maximum applicability and efficiency for future formal trials.

### 4.7 Background therapy

The clinical management of malignant tumors, particularly for locally advanced and advanced‐stage diseases, involves long‐term comprehensive treatment. The treatment regimen observed in a clinical trial represents only one component of the overall care. FLASH‑RT clinical trials should be conducted alongside appropriate background therapy. However, the impact of such treatments on the evaluation of the efficacy and safety of FLASH‐RT should be minimized as much as possible. The use of drugs that can interfere with the assessment of clinical trials should be avoided. The inappropriate use of background treatments may lead to bias.

To avoid bias, it is essential to ensure the standardization of all background treatments (including medications and rehabilitation measures) for all participants before, during, and after the trial medication period, ensuring consistency and comparability between the groups. Furthermore, in multicenter clinical trials, attention should be paid to maintaining consistency in background treatments and procedures across all trial centers. Additionally, appropriate health education and basic lifestyle recommendations should be provided.

### 4.8 Follow‐up

After the randomized treatment period, the participants were prospectively evaluated to assess the efficacy and safety of the FLASH‐RT clinical trial. Ideally, all participants should be followed up continuously to ensure the quality of the clinical trial. Malignant tumors are characterized by the dynamic and disordered proliferation of abnormal clonal cells. It is recommended that, where feasible, frequent early follow‐ups and an extended overall follow‐up period be implemented to observe short‐ and long‐term prognoses, as well as radiotherapy‐related toxicities. The duration of the trial should be designed based on the underlying mechanism of the FLASH effect: clinical trials aimed at preventing post‑FLASH‑RT recurrence and progression may require a longer follow‑up period compared to those focusing on FLASH‑RT‑related toxicities. For clinical trials investigating FLASH‐RT‐related toxicities, it is necessary to observe and measure sustained effects after the cessation of active intervention.

### 4.9 Monitoring

Participants in clinical trials should be monitored regularly. Outcome measures and adverse events should be documented and reported promptly.

### 4.10 Study outcomes/endpoint events

Research outcomes refer to toxicities, quality of life status at a specific time point, or changes in clinical and imaging findings compared to baseline. Endpoint events typically refer to the occurrence or recurrence of events within a defined time point, such as the prevention of cancer recurrence or metastasis, or the occurrence of long‐term toxicities, death, or other safety‐related events.

All primary and secondary outcomes/endpoint events, as well as the treatment regimens and durations in both the treatment and control groups (active control group), should be consistent with the trial objectives. During trial protocol design and sample size calculation, sufficient statistical power must be ensured to detect differences between treatment groups for the primary outcomes.

The efficacy endpoint measures must be clearly defined, evaluable, highly consistent, objective, and widely accepted. The primary objectives of FLASH‐RT clinical trials are to enhance tumor control, promote normal tissue protection, and improve the participants' daily living function and overall outcomes. Therefore, a dual primary endpoint or composite endpoint strategy must be adopted, and it is necessary to demonstrate non‐inferiority or superiority in antitumor efficacy, while also demonstrating a significant reduction in normal tissue toxicity. Corresponding measures can be established based on the different treatment purposes. Some measures are expressed in the form of scales that must be carefully selected and validated. Before the clinical trial commences, the consistency of the scales should be evaluated, and training on their use should be provided.

#### 4.10.1 Primary and secondary outcomes in early‐stage FLASH‐RT clinical trials

For early‐stage FLASH‐RT clinical trials, radiotherapy‐related acute toxicities should be used as primary efficacy outcomes.

- Radiotherapy‐Related Acute Toxicities. Early toxicities in predefined critical organs and tissues should be assessed using internationally recognized standards, such as CTCAE v5.0 or the RTOG/EORTC Radiation Toxicity Grading Criteria. This is central to validating the clinical translation of the FLASH effect, with a focus on early toxicities related to the treatment site that significantly impact the quality of life. Statistical design: A superiority trial design was employed to demonstrate that the incidence of early toxicities in the FLASH‐RT group was significantly lower than that in the CONV‐RT group.
- Composite Endpoint for Symptom Control and Patient‐Reported Outcomes. Definition: For trials aimed at palliative symptom relief (e.g., bone and brain metastases), the primary endpoint may be the rate of pain relief, rate of neurological function improvement, or significant enhancement in quality of life. For example, in a FLASH‐RT trial for painful bone metastases, the primary endpoint could be “significant pain relief without increased analgesic use within 3 months after treatment.” Other outcomes may also be selected as primary endpoints, depending on the treatment objective.

**Secondary outcomes include**:

- Objective Response Rate and Complete Response Rate. Proportion of patients whose tumor shrinkage met the predefined criteria according to the Response Evaluation Criteria in Solid Tumors (RECIST 1.1).
- Treatment Completion Rate and Treatment Interruption Rate. Definition: Proportion of patients who completed the entire course of radiotherapy as planned, and proportion of patients who experienced interruptions or delays in treatment due to acute toxicities.

#### 4.10.2 For Phase III or Phase IV FLASH‑RT clinical trials, the following primary efficacy endpoints are recommended

- Objective Response Rate and Complete Response Rate. The RECIST 1.1 criteria should be used to evaluate treatment response.
- Radiotherapy‑Related Late Toxicities. The core of validating the clinical translation of the FLASH effect lies in confirming its ability to reduce late toxicity through long‑term follow‑up (≥2–3 years). Assessment should prioritize toxicity events related to the treatment site, which significantly affect quality of life.
- Progression‑Free Survival. Definition: Time from randomization to disease progression or death from any cause. Advantages: It is one of the gold standards for evaluating local and regional tumor control, directly reflecting the antitumor efficacy of treatment, and can be observed earlier than overall survival. It can serve as the primary endpoint for a non‐inferiority trial to demonstrate that the tumor control effect of FLASH‐RT is not inferior to that of CONV‐RT, or as an endpoint for a superiority trial in specific tumor types where additional benefits are predicted.
- Locoregional Control Rate. Definition: Proportion of patients without progression at the primary tumor site and regional lymph nodes at a specific time point (e.g., 2 years, 3 years).
- Overall Survival. Definition: Time from randomization to death from any cause.

Advantages: It is the “gold standard” for evaluating efficacy and the hardest clinical endpoint. However, overall survival is significantly influenced by subsequent treatments and requires longer follow‐up periods and larger sample sizes. It is more suitable as a key secondary endpoint or primary endpoint in certain tumor types with poor prognosis and a lack of effective subsequent treatments.

**Secondary Endpoints**:

- Overall Survival. If not used as the primary endpoint, it must be included as the most important secondary endpoint.
- Patient‑Reported Outcomes. Definition: Systematic assessment of quality of life, symptom burden, and functional status using validated scales (e.g., EORTC QLQ‑C30 and its specific modules such as QLQ‑HN35, QLQ‑LC13, etc.).

Advantages: Evaluates treatment benefits directly from the patient's perspective, particularly by sensitively capturing improvements in the quality of life resulting from reduced toxicities. This is a key element that reflects the “patient‐centered” drug development concept.

- SMN. This represented the most severe long‐term risk. Radiotherapy itself is carcinogenic and can induce new malignancies, such as sarcomas, carcinomas, or leukemias, within or adjacent to the irradiated field, 5–10 years or even longer after treatment. This is a double‐edged sword of radiotherapy.

#### 4.10.3 Exploratory outcomes

The exploratory results may have clinical significance and are potentially related to primary or secondary endpoints. For example, exploratory studies should incorporate radiomics and blood‐based biomarkers that can predict the FLASH effect or toxicity. These include changes in urinary and bowel functions, biomarkers, and other factors.

#### 4.10.4 Economic endpoints

The economic evaluation of FLASH‐RT must adopt a societal perspective and include a long‐term dynamic analysis. Its core economic proposition lies in whether the higher upfront technological investment and potential higher cost per treatment session can be offset by the direct cost savings from a “significantly shortened treatment course” and the avoidance of substantial long‑term medical expenses and productivity losses due to “substantially reduced toxicity,” ultimately generating net socioeconomic benefits. The economic evaluation of FLASH‐RT should include both direct (medical expenses) and indirect (loss of learning or working time) costs.

- Direct Costs. The consumption of medical resources directly associated with FLASH‐RT. These mainly include:

Equipment Acquisition/Upgrading: The high cost of dedicated FLASH‐RT equipment (e.g., dedicated linear accelerators and proton FLASH‐RT systems) or the cost of upgrading existing equipment to achieve FLASH‐RT.

Supporting Infrastructure: Expenses for necessary modifications such as special shielding, power supply, and cooling systems.

Facility Construction: Costs of building new rooms or renovating existing treatment rooms to meet spatial and safety requirements.

Per‑Treatment Cost: Includes equipment depreciation, energy consumption, consumables, quality control, maintenance, etc., allocated to costs per fraction.

Personnel Costs: Additional labor costs for training physicians, physicists, and technicians in FLASH‐specific techniques (e.g., treatment planning, quality assurance, delivery).

Quality Control and Safety Costs: Extra costs arising from more complex dose verification, real‑time monitoring systems, and safety protocols under ultra‑high dose‑rate conditions.

Comparative Costs vs. CONV‐RT: The total course cost of FLASH‑RT must be compared with that of CONV‐RT (e.g., intensity‑modulated radiotherapy, proton therapy). Even if the per‑fraction cost of FLASH‐RT is higher, the total direct medical cost could be lower if the number of fractions is drastically reduced (e.g., from 30 fractions to 1–3 fractions).

Offset by Toxicity Management Costs: If FLASH‐RT significantly reduces late toxicities, it will save substantial long‑term medical expenses, including: costs of medications, hospitalization, and surgery for managing radiation pneumonitis, enteritis, and dermatitis

Long‐term costs of managing complications, such as dysphagia, nutritional support, fistulas, and osteonecrosis

- Indirect Costs. These refer to non‐medical resource consumption and productivity losses resulting from the disease and its treatment. They are often overlooked in traditional assessments, but have a significant impact on patients and society. They mainly include:

Time Lost from Work/School: CONV‐RT typically requires daily hospital visits over several weeks. If FLASH‐RT is shortened to a single day or only a few days, it could substantially reduce the need for employed patients and their accompanying family members to leave or miss work.

Transportation and Accommodation Costs:

This includes the transportation costs for patients and caregivers traveling to and from hospitals. Patients from outside the town also incur additional expenses for accommodation and living, while receiving long‐term treatment in a different city. The extremely short treatment course of FLASH‐RT could potentially eliminate the need for patients to stay away from home for an extended period.

#### 4.10.5 Adverse events

Owing to its physical characteristics of ultrahigh dose rate and extremely short irradiation time, dose monitoring is a top priority for safety. Potential adverse events related to the technical characteristics of the FLASH‐RT include the following:

- Risks Associated with Dose Delivery. Magnified consequences of dosimetric errors: Since the total dose is delivered within milliseconds, any dose deviation caused by equipment malfunction, planning error, or patient movement may be instantaneously and irreversibly “locked” into the patient, leading to severe over‑ or under‑dosage. These consequences could be more serious than those in the CONV‐RT.

Extremely High Requirements for Beam Stability: Minor fluctuations in beam intensity or position that might average out in CONV‐RT could result in nonuniform dose distribution or hot/cold spots within the target volume of FLASH‐RT.

- Risks of Biological Uncertainty. The boundary Conditions of the “FLASH Effect are not yet fully defined, and it remains uncertain whether the FLASH effect is consistent across all normal tissues and tumor types”. There may be tissues in which this protective effect is not evident, leading to unexpected toxicity. Novel biological effects of ultrahigh dose rates: There may be unrecognized mechanisms of biological damage that are unique to ultrahigh dose rates.
- Equipment and Operational Risks. New Quality Control Challenges: Existing dose measuring devices may respond differently to ultrahigh dose rates, necessitating the development of entirely new, rapid online dose verification systems. A lack of reliable quality control increases this risk. Limited Operator Experience: As new technology, the entire treatment team (physicians, physicists, and technicians) faces a steep learning curve, and the risk of operational errors may be higher in the initial stages.
- Secondary Radiation and Shielding Considerations. Ultra‑high dose‑rate irradiation may generate more secondary neutrons or scattered photons, imposing higher demands on treatment‑room shielding and the equipment itself. This poses the potential risks of occupational exposure and additional systemic doses to the patient, which require rigorous evaluation.

Comprehensive information on adverse events discovered during clinical trials should be collected, and their relationships with relevant influencing factors should be analyzed. Furthermore, a detailed analysis of adverse events and reactions should be performed based on patient‐related characteristics, baseline data (e.g., age, sex, liver and kidney function), disease severity, and concomitant medications to determine whether any adverse events reported during the trial are related to treatment.

Standard methods for collecting safety and tolerability data include spontaneous reporting records, open‐ended questions, and direct questions. The results of the active treatment and placebo should be reported separately. Detailed reporting of adverse events should follow the guidelines of local Institutional Review Boards, regulatory authorities, and clinical practice guidelines. For serious adverse events, reporting and appropriate actions should be taken within 24 hours.

## 5 Phases of trial design

### 5.1 Early‑phase clinical trials

Early phase clinical trials of FLASH‐RT primarily included Phase I and Phase I/II studies. Their core objectives are to preliminarily evaluate its safety, feasibility, and tolerability in humans and to explore suitable doses and regimens for subsequent studies. These trials typically select clinical scenarios with controllable risks and adopt a cautious, stepwise strategy.

Primary Objective (Core of Phase I): To determine the maximum tolerated or recommended Phase II dose and describe dose‐limiting toxicities.

Secondary Objectives: To assess the technical feasibility and reliability of the treatment workflow. To preliminarily observe antitumor activity (e.g., pain relief and tumor shrinkage). To explore biomarkers of the FLASH effect (e.g., specific imaging or blood parameters).

### 5.2 Exploratory trials

Exploratory clinical trials of FLASH‐RT serve as critical bridges connecting preliminary safety verifications with large‐scale confirmatory studies. These trials go beyond the preliminary question of “whether it is safe and feasible” and aim to delve deeper into the mechanism of the FLASH effect, optimal application scenarios, biomarkers, and their potential for combination with existing treatment modalities. Their design is flexible and innovative, intending to provide precise guidance for future clinical applications. The main directions and design characteristics of exploratory clinical trials in FLASH‐RT include the following:

#### 5.2.1 Core exploratory directions

- Exploration of Biological Mechanisms and Biomarkers. This is the most fundamental area of exploration, aiming to answer “why the FLASH effect occurs” and “how to predict and monitor it.”

Trial Design: Typically embedded as a translational research component in Phase I/II trials.

Exploratory Content. Blood/Tissue Biomarkers: Blood and tissue samples were collected from patients before and after treatment to analyze changes in specific cytokines, DNA damage repair markers, metabolites, or immune cell subsets, and to search for signals associated with the FLASH effect. Imaging Biomarkers: Utilizing advanced imaging techniques (e.g., functional MRI and PET‐CT) to quantitatively assess early changes in metabolism, perfusion, or fibrosis in tumors and normal tissues before and after treatment and identifying imaging features that can predict efficacy or toxicity.

“Organoid” or Patient‑Derived Xenograft Models: Constructing models using the patient's own tumor and normal tissues in vitro or in mice for parallel testing and comparing the effects of FLASH‐RT versus CONV‐RT, enabling exploration of individualized mechanisms.

- Expansion of Technical Boundaries and Indications. Advancing to more‐challenging areas based on preliminary safety data.

From “Palliative” to “Curative”: Expanding from palliative settings (e.g., bone metastases) to early‑stage, potentially curable tumors (e.g., early‑stage non‑small cell lung cancer, prostate cancer, breast cancer), exploring the potential of FLASH‐RT as a curative modality.

From “Superficial” to “Deep‑Seated/Adjacent to Critical Organs”: Expanding from extremity tumors to tumors located near critical organs in the cranium, head and neck, thorax, and abdomen. This is the key battleground for validating the protective effects of FLASH‐RT. For example, trials for brain metastases or glioblastoma should focus on monitoring the incidence of radiation necrosis, and trials for pancreatic or liver cancer should focus on protecting the intestines and liver.

Complexity of Irradiation Techniques: Exploring Volumetric Modulated Arc FLASH‐RT or Poton/Heavy‑Ion Scanning Beam FLASH‐RT to verify whether the FLASH effect can be maintained even with complex target shapes and dose distributions.

- Exploration of Combinations with New Treatment Modalities. Exploring the synergistic effects of FLASH‐RT with existing anticancer strategies, particularly immunotherapy.

FLASH‑Immunotherapy Combination: Based on the theoretical hypothesis that FLASH‐RT may uniquely effects on the tumor microenvironment and the systemic immune system (e.g., causing less lymphocyte exhaustion, potentially releasing different tumor antigens), trials are designed to combine it with immune checkpoint inhibitors. This study assessed whether this combination could stimulate stronger abscopal effects and systemic antitumor immunity.

Optimizing the Sequence of FLASH‐RT with Targeted Therapy/Chemotherapy: Exploring the optimal combination methods and sequencing with systemic therapies to maximize efficacy and manage overlapping toxicities.

- Early Assessment of Patient‑Reported Outcomes and Health Economics. Patient experience and economic data were systematically collected, even during the exploratory phase.

Patient‐reported outcomes: Standardized scales were used to conduct in‐depth assessments of the short‐ and medium‐term effects of FLASH‐RT on patient quality of life, symptom burden, and treatment experience.

Mini‑Health Economic Evaluations: Preliminarily collecting data on the number of treatment sessions, clinic visit times, costs associated with managing complications, etc., laying the groundwork for subsequent comprehensive cost‑effectiveness analyses.

#### 5.2.2 Representative design models for exploratory trials

“Basket” Design: Enroll tumors of different sites and types but using the same FLASH‐RT technology and dose regimen. This study aimed to rapidly observe the universality of the FLASH effect across different anatomical and biological environments.

“Platform” Design: Establish a flexible trial platform that allows for the rapid introduction of new combination drugs (e.g., different immunotherapies) or adjustment of technical parameters after basic safety has been verified, enabling efficient iteration.

“Randomized Phase II Screening Design”: Although the sample size is smaller than that in a Phase III trial, the patients were randomly assigned to either the FLASH‐RT group or the standard treatment group. The primary purpose is not to confirm efficacy, but to obtain preliminary evidence of efficacy signals and toxicity differences. This finding provides a decision‐making basis for determining whether large‐scale Phase III trials are warranted. This is currently the most critical exploratory trial of its kind.

### 5.3 Confirmatory trials

Confirmatory trials are conducted to further validate the preliminary data accumulated from earlier clinical trials and provide sufficient clinical evidence for new radiotherapy technologies to obtain market approval. These trials should be initiated only after the new radiotherapy technology has garnered adequate clinical, biological, and dosimetric exploration data and has shown preliminary efficacy and safety signals in the target population, with an evaluation supporting progression to this stage. Confirmatory trials are typically randomized, double‐blind, placebo‐controlled, or active comparator‐controlled superiority/non‐inferiority trials.

For FLASH‐RT, confirmatory trials primarily refer to Phase III randomized controlled clinical trials, representing the most critical, rigorous, and decisive step in the path to clinical translation. The objective is no longer to explore “whether it might be effective or safer” but to confirm, in a large‑scale population under strictly controlled conditions, whether FLASH‐RT provides a clinically meaningful net benefit to patients compared to the current standard of care. These trials are complex in design, expensive, and have long durations, making them pivotal in determining whether FLASH‐RT can become a standard treatment.

Core Hypothesis: The core hypothesis is that, while achieving equivalent or superior tumor control, FLASH‐RT can significantly reduce (superiority) damage to normal tissues, particularly late‐onset toxicities. Therefore, confirmatory trials must be capable of testing both efficacy and safety dimensions simultaneously, typically requiring the use of a composite primary endpoint or dual primary endpoints.

Key Design Considerations:

Selection of the Advantageous Population: It is essential to select tumor types and stages for which the current standard radiotherapy has clear unmet needs, especially concerning toxicity. For example, locally advanced non‐small cell lung cancer: radiation pneumonitis and cardiac toxicity are major limitations. Head and neck squamous cell carcinoma: Severe dysphagia, xerostomia, and fibrosis significantly affect the long‐term quality of life. Pelvic Malignancies (e.g., prostate cancer and cervical cancer): Toxicities affecting the rectum, bladder, and sexual function are prominent.

Control Group Setting: The control group must receive the current standard radiotherapy regimen for that specific indication (including technique, dose, and fractionation). Strict adherence to quality control standards is mandatory to ensure fair comparison.

Blinding: Due to significant differences in equipment, achieving complete blinding (for both patients and physicians) is extremely difficult, typically resulting in an open‐label design. Consequently, endpoint assessments (especially imaging evaluation and toxicity adjudication) must be performed by an independent, blinded endpoint Review Committee to minimize bias.

Long‑Term Follow‑Up: A long‑term follow‑up plan (e.g., ≥3–5 years) must be predefined to adequately assess late‐onset toxicities and survival benefits. This represents a critical window for demonstrating the value of FLASH‐RT.

## 6 KEY CONSIDERATIONS FOR STATISTICAL ANALYSIS

Statistical analysis must adhere to the general principles and standards for clinical trial data management and statistical analysis and must be strictly executed according to the statistical analysis plan described in the clinical trial protocol. For FLASH‐RT clinical trials, the importance of sample size calculations should be emphasized. Select an appropriate method for estimating the sample size based on the research objective, study design, and data type.

### 6.1 Sample size calculation

The sample size can be calculated based on findings from the literature or previous exploratory studies. To accurately calculate the sample size, researchers must follow at least four steps:

- Population standard deviation or rate should be estimated based on well‐established assumptions and meta‐analyses.
- Determine the accepted statistical error (α): The smaller the α, the larger the required sample size. Typically, α is set at 0.05.
- Determine the acceptable power of the test (1‐β): The smaller the β, the greater the power of the test. Generally, β is set at 0.1 or 0.2.
- Determine the minimum effect size to be detected (δ): Based on previous studies or pilot experiments, define a value or difference that is clinically significant. When calculating the sample size, factors such as loss to follow‐up or missing data should be considered, and the sample size should be appropriately increased based on the expected dropout rate. If the purpose of a trial is to establish non‐inferiority or equivalence, the required sample size may be larger. Imputation rules for missing data should be clearly defined in advance.

### 6.2 Selection of endpoint events and statistical methods

Evaluation of outcomes in patients receiving FLASH‐RT represents a “paradigm shift.” It requires a transition from the traditional single dimension focused primarily on “tumor control” to a multi‐dimensional comprehensive assessment encompassing “tumor control + toxicity minimization + quality of life optimization.” A successful FLASH treatment outcome should be defined as the achievement of equivalent or even superior long‐term disease control while returning to society with less suffering, faster recovery, better physical function, and improved quality of life, thereby reducing the overall disease burden on both individuals and society. All current clinical trial designs focus on scientifically and rigorously describing and validating outcomes.

For primary outcome evaluation, based on a predefined efficacy threshold, patient scores before and after follow‐up or treatment were converted into binary data. Statistical analyses were performed on these binary data to calculate the intergroup rate difference, relative risk, or odds ratio, along with their corresponding 95% confidence intervals, to evaluate treatment efficacy. Although analyzing the scores of the primary efficacy measure as continuous data using t‐tests or rank‐sum tests may yield statistically significant results in large samples, it is challenging to draw clinically meaningful conclusions based on these findings alone.

For the exploratory endpoints, appropriate data analysis methods should be selected based on the variables obtained from the trial. For example, univariate analysis may use *t*‐tests or one‐way analysis of variance (ANOVA) for quantitative data and chi‐square tests or chi‐square trend tests for categorical data. Multivariate analysis may use linear or logistic regression among other methods.

### 6.3 Subgroup analysis

If the primary endpoint analysis fails to demonstrate efficacy or a relevant association, a re‐stratified or subgroup analysis based on factors such as disease severity or research focus may yield clinically significant findings. However, the results of exploratory subgroup analyses (e.g., disease severity and age group) should serve only as a reference for designing future clinical trials. Subgroup analyses were planned before completion of the study design.

### 6.4 Covariate analysis

Certain baseline characteristics such as disease severity and age may be considered as covariates for analysis.

### 6.5 Applicable data analysis methods

- The Intention‐to‐Treat (ITT) Analysis. All the participants are randomized into groups and should be analyzed as members of their assigned groups. For cases with incomplete treatment course data, the last observation carried forward method should be used to impute data for the final outcome, and efficacy should be analyzed based on the intention‐to‐treat principle.
- Per‐Protocol (PP) Analysis. Statistical analysis of efficacy is performed for all patients who strictly adhere to the study protocol, demonstrate good compliance, do not take prohibited medications during the trial, and complete all the required entries in the Case Report Forms.
- Safety Data Analysis. All patients who undergo FLASH‐RT after randomization and have at least one safety assessment should be included. The analysis focuses on the occurrence of adverse events and reactions, as well as normal and abnormal changes in vital signs and laboratory indicators.

## 7 EXPLANATION

This expert consensus was jointly developed through discussions between experts from the China Anti‐Cancer Association Radiation Oncology Committee and the Chinese Medical Doctor Association Radiation Oncology Physician Committee. It aims to provide methodological guidance for the design, implementation, and evaluation of FLASH‐RT clinical trials to assess the efficacy and safety of FLASH‐RT through standardized clinical trials and offer evidence to support its clinical translation and application.

### 7.1 Applicability of this expert consensus

This expert consensus is applicable to the FLASH‐RT clinical trials conducted on patients at all stages. The definition of FLASH‐RT can be found in Section “4.1.1”.

FLASH‐RT products are those developed and registered for marketing in accordance with relevant regulations, such as the “Regulations on the Supervision and Administration of Medical Devices,” the “Law on the Prevention and Control of Radioactive Pollution,” and the “Basic Standards for Ionizing Radiation Protection and Radiation Source Safety.” The equipment (new or significantly modified) used to deliver the FLASH treatment must be managed as a Class III medical device. Medical institutions conducting FLASH‐RT trials must hold a valid “Medical Institution Practice License,” and their scope of practice must include “radiation oncology for tumors.” All personnel involved (radiation oncologists, medical physicists, and radiation therapists) must possess the corresponding professional qualification certificates and have received specialized training in FLASH‐RT technology.

### 7.2 Non‐binding nature and reference use

This expert consensus applies to the research and development of FLASH‐RT technology and is intended only for recommendatory guidance. When applying this consensus, the interpretation should first be based on the general requirements of clinical trials. It should also be used in conjunction with references to good clinical practices (GCP) and other relevant technical guidelines issued domestically or internationally. This expert consensus is advisory and does not serve as a mandatory requirement for the market registration of new radiotherapy devices.

### 7.3 Role of the researcher and individualized trial design

This expert consensus cannot replace the responsibility of investigators in designing FLASH‐RTs tailored to the characteristics of specific diseases that reflect their mechanisms. Researchers should develop a well‐structured clinical trial protocol based on the physicochemical properties and clinical positioning of the FLASH‐RT under study, informed by preclinical findings, advances in the relevant field, and clinical practice, while adhering to GCP requirements with a scientific and rigorous approach.

### 7.4 Dynamic nature of the consensus

Given the rapid advances in FLASH‐RT research and the continuous improvement in disease treatment modalities alongside the evolution of medical science and clinical practice, evaluation methods for clinical trial designs in FLASH‐RT will also be updated accordingly. Therefore, the expert consensus represents only the current recommendations. Should more scientifically sound and generally accepted methodologies emerge with the advancement of medical science, they may also be adopted in clinical trials, provided relevant supporting evidence is available. This expert consensus will also be continuously refined and updated based on scientific research progress.

## 8 SUMMARY

FLASH‐RT clinical trials have a significant value in overcoming the limitations of normal tissue damage associated with CONV‐RT. During the drafting and preparation of the Expert Consensus on Clinical Trial Design Guidelines for FLASH Radiotherapy, the principles of extensive verification, broad participation, and wide consultation were followed. However, this expert consensus may still have certain limitations. We hope to continuously improve this through feedback from all the parties to ensure its applicability at all stages.

## CONFLICTS OF INTEREST STATEMENT

Jinming Yu is the Editor‐in‐Chief of the journal and co‐author of this article. He was excluded from the peer review process and from all editorial decisions related to the acceptance and publication of this article. Peer review was handled independently by the other editor to minimize bias.

## ETHICS STATEMENT

The authors are accountable for all aspects of this work and ensure that questions related to the accuracy or integrity of any part are appropriately investigated and resolved.
